# Insights into the processes that drive the evolution of drug resistance in *Mycobacterium tuberculosis*

**DOI:** 10.1111/eva.12654

**Published:** 2018-06-21

**Authors:** Quang Huy Nguyen, Lucie Contamin, Thi Van Anh Nguyen, Anne‐Laure Bañuls

**Affiliations:** ^1^ Department of Pharmacological, Medical and Agronomical Biotechnology University of Science and Technology of Hanoi Vietnam Academy of Science and Technology (VAST) Hanoi Vietnam; ^2^ Institute of Research for Development UMR MIVEGEC (CNRS‐IRD‐University of Montpellier) Montpellier France; ^3^ LMI Drug Resistance in South East Asia (LMI DRISA) University of Science and Technology of Hanoi Vietnam Academy of Science and Technology (VAST) Hanoi Vietnam; ^4^ Department of Bacteriology National Institute of Hygiene and Epidemiology (NIHE) Hanoi Vietnam

**Keywords:** compensatory mutation, drug resistance mutation, epistasis, evolution, fitness cost, multidrug‐resistant tuberculosis, mycobacterium tuberculosis

## Abstract

At present, the successful transmission of drug‐resistant *Mycobacterium tuberculosis*, including multidrug‐resistant (MDR) and extensively drug‐resistant (XDR) strains, in human populations, threatens tuberculosis control worldwide. Differently from many other bacteria, *M. tuberculosis* drug resistance is acquired mainly through mutations in specific drug resistance‐associated genes. The panel of mutations is highly diverse, but depends on the affected gene and *M. tuberculosis* genetic background. The variety of genetic profiles observed in drug‐resistant clinical isolates underlines different evolutionary trajectories towards multiple drug resistance, although some mutation patterns are prominent. This review discusses the intrinsic processes that may influence drug resistance evolution in *M. tuberculosis,* such as mutation rate, drug resistance‐associated mutations, fitness cost, compensatory mutations and epistasis. This knowledge should help to better predict the risk of emergence of highly resistant *M. tuberculosis* strains and to develop new tools and strategies to limit the development and spread of MDR and XDR strains.

## 1 INTRODUCTION

Tuberculosis (TB), an infectious airborne disease mainly caused by *Mycobacterium tuberculosis*, is one of the world's deadliest infectious diseases. In 2015, TB affected approximately 10.4 million people and killed 1.8 million of them (WHO, 2016). The current recommended treatment for new patients with drug‐susceptible TB is a 6‐month regimen using a combination of four‐first‐line anti‐TB drugs: isoniazid, rifampicin, ethambutol and pyrazinamide (WHO, 2016). This effective regimen was developed in the early 1970s and showed a high cure rate, higher than 98%, in clinical trials (STS/BMRC, 1988). The regimen has not been changed, but now, its global treatment success rate is of 83% for new patients with TB (WHO, 2016). Indeed, its use for almost five decades has led to the emergence of first‐line drug resistance. Multidrug‐resistant (MDR, Box 1) TB is the most problematic first‐line drug‐resistant form (Nachega & Chaisson, 2003). The treatment of MDR TB requires second‐line drugs that are more expensive and toxic than first‐line drugs. Nevertheless, during the last 15 years, patients with extensively drug‐resistant (XDR, Box 1) TB have been reported in 105 countries. In 2015, 480,000 patients with MDR TB were reported worldwide and approximately 10% of them developed XDR TB (WHO, 2016).

BOX 1 **Glossary**

1 *Acquired resistance*: the ability of a bacterial population to resist the activity of a particular drug to which it was previously susceptible.

*Biological fitness*: the capability of an individual with a certain genotype to reproduce and survive in a competitive environment.

*Clonal interference*: competition between lineages (“clones”) arising from different beneficial mutations to reach the fixation in asexual organisms.

*Compensatory mutation*: a second‐site mutation acquired that arises after the acquisition of resistance mutation and that lessens or alleviates the fitness cost associated with the acquisition of the resistance‐associated mutation.

*Cross‐resistance*: the acquisition by a microbe of resistance to one drug through direct exposure and the gain, in parallel, of resistance to one or more other drugs to which it has not been exposed.

*Epistasis*: a form of interaction between genes or mutations that influences a phenotype. Epistasis occurs when the combined fitness effect of multiple alleles from same locus or different loci is different from the sum of the individual allele effects.

*Extensively drug resistance (XDR)*: MDR (see below) *Mycobacterium tuberculosis* also resistant to at least one of the three‐second‐line injectable drugs (kanamycin, amikacin and capreomycin) and one fluoroquinolone (ofloxacin, levofloxacin, moxifloxacin or gatifloxacin).

*Fitness cost of resistance mutation*: a decrease in the relative fitness of the drug‐resistant mutants in comparison with their drug‐susceptible counterparts.

*Genetic drift*: random changes in the frequency of alleles over time usually in small populations.

*Innate resistance*: the innate ability of a bacterial species to resist activity of a particular drug.

*Linkage disequilibrium*: the nonrandom association of alleles at different loci.

*Multidrug resistance (MDR): Mycobacterium tuberculosis* resistant at least to isoniazid and rifampicin, the two more potent fist‐line drugs.

*Mutation rate*: the number of mutations per nucleotide site (bp) per generation. It is worth noting that in the case of antibiotic resistance, the mutation rate is frequently defined as the rate of resistance acquisition (see below).

*Negative epistasis*: if the cost of double mutant in the absence of antimicrobial use is higher than the total cost of each resistance determinant on its own.

*Parallel evolution*: evolution of a similar trait in closely related, independently evolving lineages.

*Positive epistasis*: if the cost of double mutant in the absence of antimicrobial use is smaller than the total cost of each resistance determinant on its own.

*Purifying selection*: selection reducing the frequency of deleterious alleles in a population.

*Rate of resistance acquisition*: the *in vitro* frequency at which detectable mutants arise in a bacterial population in the presence of a given antibiotic concentration.

*Mycobacterium tuberculosis* is a highly clonal bacteria (absence of recombination) with an extremely conserved genome and a long history of co‐evolution with humans (Bañuls, Sanou, Nguyen, & Godreuil, 2015; Comas et al., 2013). At present, *M. tuberculosis* consists of seven phylogenetic lineages associated with particular geographic regions and differing, among others, in virulence, biological fitness and propensity to acquire drug resistance (Comas et al., 2013; Merker et al., 2015; Stucki et al., 2016; Warner, Koch, & Mizrahi, 2015). Besides the lineage‐specific biological characteristics, the remarkable capacity of adaptation and the variety of extrinsic and intrinsic processes contribute specifically to the emergence and spread of highly drug‐resistant strains (Coscolla & Gagneux, 2014; Müller, Borrell, Rose, & Gagneux, 2013; Trauner, Borrell, Reither, & Gagneux, 2014; Warner et al., 2015).

As example of intrinsic mechanisms, epistasis (Box 1), which plays an important role in the evolution of organisms in general, is also known to drive the evolution of antibiotic resistance (Lehner, 2011). Epistasis can occur between mutations in the same gene or in different genes and can lead to negative or positive effects (Box 1) (Lehner, 2011; Wong, 2017). This mechanism may generate the combination of a set of alleles from different loci, also called linkage disequilibrium (Box 1). The spread of these sets of co‐adapted alleles in the population is then favoured by the clonal reproductive mode of *M. tuberculosis*. Regarding the drug resistance, many studies underline that epistatic interactions can occur between different drug resistance mutations, between drug resistance mutations and compensatory mutations and/or the genetic background of the organism (Borrell et al., 2013; Lehner, 2011; Trindade et al., 2009; Wong, 2017).

In this review, we focus on the intrinsic factors influencing the drug resistance evolution in *M. tuberculosis*, particularly the mutation rate, drug resistance‐associated mutations, fitness cost of resistance mutations, compensatory mutations and epistasis (Box 1). An understanding of the role of these intrinsic factors is essential to get insights into the evolutionary trajectories of drug resistance in *M. tuberculosis* and to help identifying the best strategies to control the emergence and spread of highly drug‐resistant strains.

## 2 MUTATION RATE AND DRUG RESISTANCE ACQUISITION

*Mycobacterium tuberculosis* is characterized by a low mutation rate (about 2 × 10^−10^ mutations/bp/generation) (Ford et al., 2011), with an estimated evolutionary rate of 0.4—0.5 single nucleotide polymorphisms (SNPs)/genome/year and a divergence rarely higher than five SNPs in 3 years (Roetzer et al., 2013; Walker et al., 2013). Despite this low mutation rate, the number of drug resistant, especially MDR and XDR TB cases, due to the acquisition of mutations, is progressively increasing worldwide.

Besides innate drug resistance mechanisms (for instance, the specific characteristics of the cell envelope of *M. tuberculosis* and the active drug efflux mechanism) (Sarathy, Dartois, & Lee, 2012), chromosomal mutations are the major mechanism of drug resistance acquisition in *M. tuberculosis* (Table 1) (Sandgren et al., 2009; Zhang & Yew, 2015). The rate for evolution of drug resistance to major first‐ and second‐line drugs ranges from 10^−6^ to 10^−10^ mutations/bacterial cell/generation (McGrath, Gey van Pittius, van Helden, Warren, & Warner, 2014). This rate might also be affected by the drug concentration in the medium, the drug resistance profile of the strain and its genetic background (Ford et al., 2013; McGrath et al., 2014).

**Table 1:** Modes of action of the main first‐ and second‐line anti‐TB drugs, mechanisms of drug resistance and mutation frequency for each gene in clinical *Mycobacterium tuberculosis* isolates

Group	Drug	Drug action	Drug resistance‐associated gene(s)	Mutation frequency in clinical isolates (%)^a^
First‐line anti‐TB drugs	Rifampicin	Binding to the β‐subunit of the RNA polymerase, inhibition of the elongation of messenger RNA	*rpo*B encoding for β‐subunit of RNA polymerase	90–100
	Isoniazid	Activation by a catalase‐peroxidase enzyme	*kat*G encoding for catalase‐peroxidase	40–97
		Inhibition of the synthesis of mycolic acids through binding to NADH‐ACP‐reductase	*inh*A encoding for fatty acid enoyl acyl carrier protein reductase A (InhA)	8–64
	Ethambutol	Inhibition of an arabinosyl transferase involved in cell wall synthesis	*emb*B encoding for arabinosyl transferase	47–89
	Pyrazinamide	‐ Activation by the pyrazinamidase ‐ Disruption of membrane energetics that inhibits membrane transport	*pnc*A encoding for pyrazinamidase	44–97
	Streptomycin	Inhibition of protein synthesis by interaction with the 16S rRNA and the S12 ribosomal protein	*rrs* encoding for 16S rRNA subunit	12–26
			*rps*L encoding for S12 ribosomal protein	40–68
		Inhibition of methylation of 16S rRNA	*gid*B encoding for 7‐methylguanosine methyltransferase	5–13
Second‐line anti‐TB drugs	Amikacin, kanamycin, capreomycin	Inhibition of protein synthesis by interaction with the 16S rRNA	*rrs* encoding for 16S rRNA	40–90
	Kanamycin	Inhibition of acetyltransferase	*eis* encoding for aminoglycoside acetyltransferase	28–80
	Capreomycin	Inhibition of methylation of 16S rRNA & 23S rRNA	*tly*A encoding for 2′‐O‐methyltransferase	4–13
	Ofloxacin, levofloxacin, moxifloxacin, gatifloxacin	Inhibition of the topoisomerase II (DNA gyrase) lead to the inhibition of DNA supercoiling	*gyr*A encoding for DNA gyrase subunit A and	70–90
			*gyr*B encoding for DNA gyrase subunit B	0–11
	Ethionamide	Inhibition of the synthesis of mycolic acids by interaction with NAD that inhibits the enoyl‐ACP reductase	*inh*A encoding for fatty acid enoyl acyl carrier protein reductase A (InhA)	33–62
		Inhibition of metabolic activation by interaction with the transcriptional repressor of the Monooxygenase (EthA)	*eth*A encoding for EthA	46–72
			*eth*R encoding for transcriptional repressor EthR, NADH‐ACP	0–4

*Note*

a See the following papers for details (Campbell et al., 2011; Ramirez‐Busby & Valafar, 2015; Sandgren et al., 2009; Vilcheze & Jacobs, 2014; Zhang & Yew, 2015).

As the genes responsible for resistance to the various anti‐TB drugs are generally not functionally related, the risk of emergence of spontaneous double, triple and quadruple drug‐resistant mutants is theoretically extremely low, ranging from about 10^−14^ mutants (for isoniazid and rifampicin) to 10^−25^ mutants per population (for isoniazid, rifampicin, ethambutol and pyrazinamide). Furthermore, clinical data estimated that the population size in active pulmonary disease ranges between 10^7^ and 10^10^ bacilli (Nachega & Chaisson, 2003), thus the risk of spontaneous drug resistance‐associated mutations should be very low. In addition, drug resistance‐associated mutations may impose a fitness cost because they target essential cell biological functions (Melnyk, Wong, & Kassen, 2015). Therefore, in theory the chance of drug resistance acquisition should be negligible when the four effective first‐line drugs are used in combination. However, by mathematical modelling, Colijn, Cohen, Ganesh, & Murray (2011) estimated that the probability of acquisition of resistance to both isoniazid and rifampicin is as high as 10^−4^ to 10^−5^ mutants/bacterial population. Similar to that, in a clinical study, Gao et al. (2016) found that 3.7% (62/1671) of pan‐susceptible clinical isolates (susceptible to isoniazid, rifampicin, streptomycin and ethambutol) acquired different resistance patterns during the standard short‐course chemotherapy according to the Directly Observed Treatment (DOT) guidelines. Among the 62 strains with acquired drug resistance, approximately 10% were resistant to four drugs, 22.6% to three drugs, 21% to two drugs and the remaining 46.8% were mono‐drug resistant. These data underline that multiple drug resistance acquisition emerges at higher rate under strong drug selection pressure than theoretically predicted.

Regarding the genetic background, Ford et al. (2013) demonstrated that overall, the mutation rates for drug resistance acquisition are higher in *M. tuberculosis* lineage 2 (East Asia, mainly Beijing strains) than in lineage 4 (Euro‐American). In addition, the authors also demonstrated that the risk of de novo MDR acquisition before treatment is higher (approximately 22‐fold) in macaques infected with *M. tuberculosis* strains of lineage 2 than in animals infected with lineage 4 strains. These data are consistent with the high drug resistance potential of lineage 2 observed in many epidemiological studies (Casali et al., 2014; Merker et al., 2015). Besides the effect of genetic background, Ford et al. (2013) also showed that differences in target size (defined as the number of resistance‐associated mutations) contribute to the two‐ to thirty‐five‐fold differences in rifampicin resistance rates that they have measured in their samples.

It is worth noting that extrinsic factors such as the economic and social situation of individuals or populations, the major political events (e.g., the fall of the Soviet Union) and the quality of TB control programmes also strongly influence the speed of drug resistance spread (Eldholm et al., 2016; Klopper et al., 2013; Müller, Chihota, et al., 2013). In vivo, the drug resistance acquisition also greatly varies depending on the location of bacterial populations in the body and the characteristics of the drugs (Kempker et al., 2015; Warner et al., 2015). Indeed, even under an optimal treatment, the bacteria can be exposed to suboptimal drug concentrations due to variable degrees of tissue penetration linked to the tissue and/or drug. In particular, the poor penetration ability of drugs in cavitary lesions is an important risk factor for the emergence of drug resistance in TB patients under long‐term treatment course (Kempker et al., 2015). Furthermore, using mathematical modelling, Moreno‐Gamez et al. (2015) demonstrated that the imperfect drug penetrance leads to spatial mono‐therapy and thus to a rapid evolution towards MDR. In addition, variations in drug absorption in patients (pharmacokinetic variability) can be also a factor of emergence of MDR TB (Pasipanodya & Gumbo, 2011).

## 3 INTRA‐HOST GENETIC VARIABILITY OF DRUG‐RESISTANT POPULATIONS

**Figure 1:**
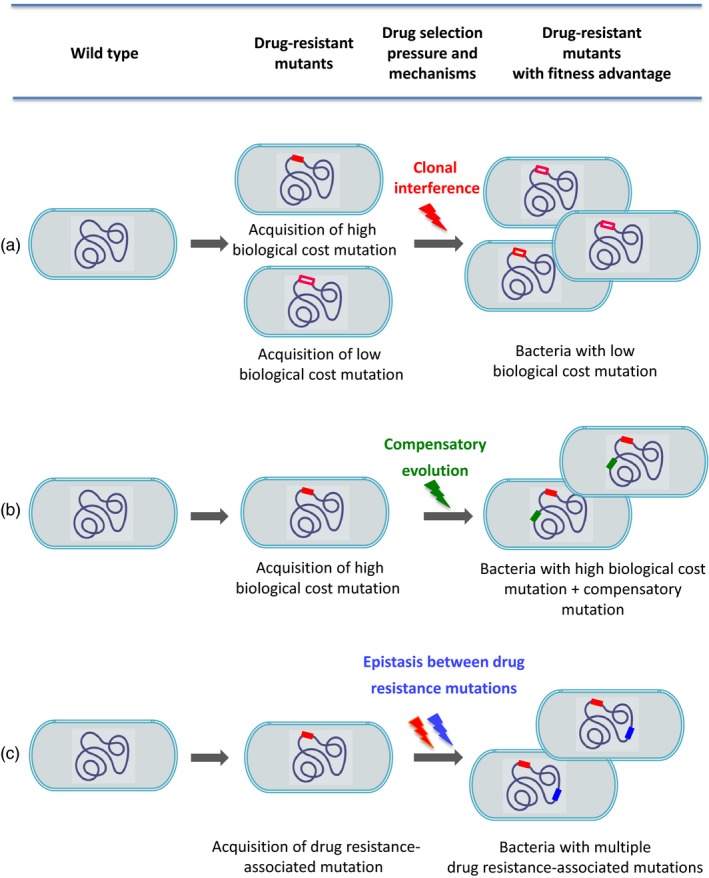
Origin and evolution of drug resistance in *Mycobacterium tuberculosis* (modified from (Chang et al., 2015; Hughes & Andersson, 2015)). The figure represents the evolution of bacteria from wild type to drug‐resistant mutants with fitness advantage and illustrates several different mechanisms of fitness increase. (a) Wild type can acquire different drug resistance‐associated mutations in a same gene with high or low biological cost. The bacteria with low biological cost mutations will be selected under drug pressure by clonal interference and will propagate. (b) Under drug pressure, positive epistasis may favour the acquisition of compensatory mutations to alleviate the fitness cost exerted by certain drug resistance‐associated mutations. (c) Driven by positive epistasis, drug‐resistant mutants are likely to be more prone to accumulate drug resistance‐associated mutations at higher frequencies (Nguyen, Nguyen, et al., 2017; Trindade et al., 2009)

The intra‐host evolution of bacterial resistance patterns is one of the key aspects of drug resistance emergence and spread (Eldholm et al., 2014; Meacci et al., 2005; Merker et al., 2013). The existence of genetically variable drug‐resistant bacterial populations within a single patient is now acknowledged and could affect drug resistance evolution (Black et al., 2015; Eldholm et al., 2014; Müller, Borrell, et al., 2013; Shamputa et al., 2004). Meacci et al. investigated *M. tuberculosis* population evolution in a noncompliant patient during more than 12 years of active disease. They identified the emergence of a MDR *M. tuberculosis* population from one single parental strain that was composed of discrete subpopulations with different drug resistance‐associated gene variants (Meacci et al., 2005). This suggests that the intra‐host bacterial population evolved over time by acquiring and accumulating different gene mutations associated with resistance to isoniazid, rifampicin and streptomycin. This led to the emergence, in one single patient, of different coexisting populations that harbour different drug susceptibility profiles. In another study, Sun et al. (2012) described the dynamic changes of the drug resistance‐associated mutation profile in *M. tuberculosis* populations at different stages of drug resistance acquisition. These authors found four to five transient drug resistance mutants in the same sputum sample, but only the fittest resistant mutant became fixed over time. Similar to that, Eldholm et al. (2014) monitored the evolution of an XDR strain from a susceptible ancestor in a single patient. They showed that drug resistance‐associated mutations were acquired multiple times by individual clones, but only one expanded and replaced the other clones. In an ultimate manner, adaptive mutants are fixed and become dominant while others are lost by competition, referred as clonal interference (Box 1, Figure 1a) (Gerrish & Lenski, 1998). In addition, recent studies also demonstrated that *M. tuberculosis* populations can evolve measurably in response to selection pressures imposed by the environment within hosts (Lieberman et al., 2016; O'Neill, Mortimer, & Pepperell, 2015). This process can lead to the spatial structuring of the bacterial population within host (lungs) into related subpopulations that will evolve independently (parallel evolution, Box 1) as demonstrated previously (Gygli, Borrell, Trauner, & Gagneux, 2017). All these studies underline the constant genome evolution due to the acquisition of multiple independent mutations in the bacterial population despite the evolutionary bottleneck imposed by purifying selection (Box 1) due to drug selective pressure and clonal interference (Figure 1a).

## 4 CHARACTERISTICS AND DIVERSITY OF DRUG RESISTANCE‐ASSOCIATED MUTATIONS

The mutation frequency and type vary in function of different parameters, such as the geographic region, the drug resistance pattern and genetic background (Fenner et al., 2012; Hillemann, Kubica, Rusch‐Gerdes, & Niemann, 2005; Lipin, Stepanshina, Shemyakin, & Shinnick, 2007; Qian et al., 2002). Despite the large diversity of mutation patterns globally, only few specific mutations are predominant (Table 2) (Sandgren et al., 2009; Zhang & Yew, 2015). For instance, in the case of rifampicin resistance, hundreds of *rpo*B mutations have been described (not all were associated with rifampicin resistance), but more than 80% of rifampicin‐resistant isolates display mutations in three codons *rpo*B531, 526 and 516 (Campbell et al., 2011; Lipin et al., 2007; Pozzi et al., 1999; Telenti et al., 1993). Similar to that, among the approximately 300 mutations found in the *kat*G gene, the prevalence of *kat*G S315T mutation can vary between 32% and 95% in isoniazid‐resistant clinical isolates depending on the geographic regions and drug resistance patterns (Hazbon et al., 2006; Lipin et al., 2007; Mokrousov et al., 2002; Vilcheze & Jacobs, 2014).

**Table 2:** The most frequent drug resistance‐associated mutations found in clinical drug‐resistant *Mycobacterium tuberculosis* isolates, including MDR and XDR samples

Drug(s)	Drug resistance‐associated gene(s)	Frequent mutation (amino acid/nucleotide change)	Mutation frequency in clinical drug‐resistant isolates (%)^b^
Isoniazid	*kat*G	315 (Ser‐Thr)	32–95
	*inh*A	‐15 (C‐T)^a^	8–71
Rifampicin	*rpo*B	531 (Ser‐Leu)	41–74
		526 (His‐Tyr)	6–24
		526 (His‐Asp)	2–30
		516 (Asp‐Val)	5–18
Streptomycin	*rps*L	43 (Lys‐Arg)	35–62
		88 (Lys‐Arg)	13–28
	*rrs*	514 (A‐C)^a^	3–12
Ethambutol	*emb*B	306 (Met‐Val)	40–60
Fluoroquinolones	*gyr*A	94 (Asp‐Gly)	25–60
		90 (Ala‐Val)	12–30
Kanamycin, amikacin and capreomycin	*rrs*	1401 (A‐G)^a^	30–90

*Notes*

a Nucleotide change.

b See the following studies for reference (Campbell et al., 2011; Duong et al., 2009; Hazbon et al., 2006; Hillemann et al., 2005; Lipin et al., 2007; Mokrousov et al., 2002; Müller et al., 2011; Niehaus et al., 2015; Perdigao et al., 2010; Pozzi et al., 1999; Qian et al., 2002; Shi et al., 2011; van Soolingen et al., 2000; Sreevatsan et al., 1996, 1997; Telenti et al., 1993; Von Groll et al., 2009).

In addition, different mutations in the same gene or in different genes can produce similar drug resistance phenotypes (Sandgren et al., 2009; Zhang & Yew, 2015), but can be associated with similar or different drug resistance levels (Fenner et al., 2012; Gagneux, Long, et al., 2006; Huitric, Werngren, Jureen, & Hoffner, 2006). For instance, some mutations in *rpo*B gene, such as *rpo*B S531L, H526Y, H526D and H526R, are often associated with high levels of rifampicin resistance, while mutations including *rpo*B L511P, H526L, H526N, L533P and I572F are generally linked to low levels of rifampicin resistance (Huitric et al., 2006; Van Deun et al., 2009). Similar to that, *kat*G mutations are often associated with high levels of isoniazid resistance, whereas *inh*A mutations with low levels (Fenner et al., 2012; van Soolingen et al., 2000; Vilcheze & Jacobs, 2014).

Furthermore, mutations in different regions of the same gene can be associated with different drug resistance phenotypes. For instance, mutations in the 530 loop and 915 region of *rrs* gene are associated with streptomycin resistance (Sreevatsan et al., 1996), while mutations in the 1400—1500 region are linked to resistance to kanamycin, amikacin and capreomycin (Jugheli et al., 2009). In particular, cross‐resistance phenomena (Box 1) have also been described in *M. tuberculosis* (Andries et al., 2014; Jugheli et al., 2009; Vilcheze & Jacobs, 2014).

At last, the mutation type has been correlated with the genetic background of *M. tuberculosis* lineages in several studies (Hillemann et al., 2005; Mokrousov et al., 2006; Qian et al., 2002; Ribeiro et al., 2014). For instance, the *kat*G S315T is prevalent in lineage 2, conversely the *inh*A‐15 mutation is associated mostly with lineage 1 (Casali et al., 2014; Fenner et al., 2012; Gagneux, Burgos, et al., 2006; Mokrousov et al., 2002; Nguyen, Nguyen, et al., 2017). Similar to that, the *rpo*B S531L mutation is observed mainly in lineage 2 compared with other lineages, while the *rpo*B D516V mutation is more frequent in lineage 4 (LAM family) (Casali et al., 2014; Hillemann et al., 2005; Lipin et al., 2007). In particular, the prevalence of specific drug resistance‐associated mutations also varies within the lineage, such as the frequencies of the *rpo*B S531L and *kat*G S315T mutations are greater in the modern (typical) Beijing strains than in ancient (atypical) ones (Hillemann et al., 2005; Lipin et al., 2007; Qian et al., 2002). These differences could be the result of epistatic interactions (see Box 1 and below) and might reflect the adaptation of *M. tuberculosis* sublineages to the different human populations and the efficiency of treatment and public health strategies (Bañuls et al., 2015; Comas et al., 2013; Eldholm et al., 2016).

## 5 FITNESS COST OF DRUG RESISTANCE‐ASSOCIATED MUTATIONS

As the drug targets are generally involved in important biological functions, mutations in the genes encoding these factors should impart a biological cost that leads to reduced fitness of the resistant strains in comparison with the sensitive ones, in the absence of antibiotics (Melnyk et al., 2015). According to that, several studies showed that drug‐resistant *M. tuberculosis* mutants are characterized by reduced fitness (Billington, McHugh, & Gillespie, 1999; Gagneux, Long, et al., 2006; Mariam, Mengistu, Hoffner, & Andersson, 2004). However, the extent of the biological cost depends on the mutation and the strain genetic background (Billington et al., 1999; Bottger, Springer, Pletschette, & Sander, 1998; Gagneux, Long, et al., 2006; Pym, Saint‐Joanis, & Cole, 2002). Furthermore, in the absence of genetic drift (Box 1), drug‐resistant mutations with low or no biological cost are more likely to be selected and maintained in the populations (Farhat et al., 2013; Osorio et al., 2013). For instance, the predominant mutations associated with high level of drug resistance and a low or no biological cost, such as *kat*G S315T, *rpo*B S531L, *rps*L K43R and *gyr*A D94G (conferring resistance to isoniazid, rifampicin, streptomycin and fluoroquinolones respectively), are more frequently found in clinical drug‐resistant isolates (Billington et al., 1999; Bottger et al., 1998; Campbell et al., 2011; Casali et al., 2014; Gagneux, Burgos, et al., 2006; Gagneux, Long, et al., 2006; Mariam et al., 2004; Pym et al., 2002). Indeed, some of these resistance mutations do not reduce bacterial fitness in the absence of treatment (Bergval, Schuitema, Klatser, & Anthony, 2009; Huitric et al., 2006; Mariam et al., 2004). It is worth noting that MDR and XDR strains associated with outbreaks often carried these mutations explaining the successful spread of these highly drug‐resistant strains in the community (Casali et al., 2014; Cohen et al., 2015; Niehaus, Mlisana, Gandhi, Mathema, & Brust, 2015; de Vos et al., 2013). All these observations suggest that the prevalent mutations in clinical isolates have been positively selected because of their low biological cost (Figure 1a) (Farhat et al., 2013; Osorio et al., 2013). As demonstrated by Bergval et al. (Bergval et al., 2009), drug resistance mutation patterns of in vitro selected‐resistant mutants do not always reflect mutation profiles obtained in clinical isolates. Indeed, mutations associated with high biological cost of resistance detected in in vitro drug‐resistant mutants are rarely found in clinical drug‐resistant isolates (Bergval et al., 2009; Gagneux, Long, et al., 2006; Huitric et al., 2006). For example, mutations in the *kat*G gene that lead to complete loss of the catalase‐peroxidase enzyme function (conferring resistance to isoniazid), such as frame‐shift nucleotide deletions or insertions, are found less frequently in clinical than in in vitro mutants (Bergval et al., 2009). Several reasons can explain the different mutation patterns obtained in in vitro mutants and clinical isolates, such as the long evolution within the human body, the clonal interference, the variability in drug pressure and the parallel evolution.

The biological cost of pyrazinamide resistance mutations is of interest to investigate the evolution of pyrazinamide resistance in *M. tuberculosis*. The high diversity of mutations in the *pnc*A gene detected in clinical isolates (Ramirez‐Busby & Valafar, 2015) can be explained by two different hypotheses. First, *pnc*A mutations associated with pyrazinamide resistance might not cause any fitness deficit. Indeed, *pnc*A seems to be nonessential because *M. tuberculosis* can survive without this gene using other metabolic pathways (Martinez, Holmes, Jelfs, & Sintchenko, 2015; Stoffels, Mathys, Fauville‐Dufaux, Wintjens, & Bifani, 2012). Therefore, each mutation can have the same probability to be selected and transmitted. In an alternative way, *pnc*A mutations could be associated with high cost of resistance that impairs *M. tuberculosis* transmission (den Hertog, Sengstake, & Anthony, 2015). This hypothesis is supported by the lack of *pnc*A mutant clusters, thus reflecting a low transmission potential. However, clusters of *pnc*A mutants have been described in some specific MDR and XDR *M. tuberculosis* outbreaks in South Africa and in Argentina showing the successful transmission of these PZA‐resistant clones (Cohen et al., 2015; Eldholm et al., 2015; Müller, Chihota, et al., 2013). The development of experimental evolution studies will allow assessing the biological cost magnitude of *pnc*A mutations.

Several works investigated the link between *M. tuberculosis* genetic background and the cost of drug resistance mutations. Gagneux, Long, et al. (2006) found differences in biological cost for the rifampicin resistance‐associated *rpo*B H526D mutation between the lineages 2 and 4, while the *rpo*B S531L mutants showed similar costs in both lineages. The *inh*A‐15 and *kat*G S315T mutations are strongly associated with lineage 1 and modern lineages, respectively (Casali et al., 2014; Fenner et al., 2012; Gagneux, Burgos, et al., 2006). On the contrary, *kat*G mutations other than *kat*G S315T that likely abrogate enzyme activity result in high biological cost and seem to be more associated with lineage 2 (Gagneux, Burgos, et al., 2006). Thus, lineage 2 could be better adapted to compensate for the loss or reduced activity of this catalase‐peroxidase enzyme in the context of isoniazid resistance. This hypothesis could also explain why the Beijing strains are generally strongly associated with resistance to isoniazid, regardless of the type of *kat*G resistant‐associated mutations and country (Fenner et al., 2012; Gagneux, Burgos, et al., 2006; Mokrousov et al., 2002; Ribeiro et al., 2014; van Soolingen et al., 2000).

## 6 COMPENSATORY MUTATIONS

Compensatory mutations can alleviate the loss of fitness produced by drug resistance‐associated mutations (Figure 1b) (Bottger et al., 1998; Brandis, Wrande, Liljas, & Hughes, 2012). In *M. tuberculosis,* data on compensatory mutations are still limited and mainly focused on isoniazid and rifampicin resistance (Comas et al., 2012; Sherman et al., 1996; Song et al., 2014; de Vos et al., 2013). Nevertheless, mechanisms of compensatory evolution were also proposed for other drug resistance genotypes (Table 3).

**Table 3:** Mechanisms of drug resistance, fitness costs and compensatory mechanisms in *Mycobacterium tuberculosis*

Genetic mutation(s)	Mechanism of resistance	Fitness cost	Compensatory mechanism
*kat*G	Reduced prodrug activation	Reduced protection against oxidative damage	Overexpression of *ahp*C by mutations in its promoter
*inh*A promoter	NADH‐ACP‐reductase overexpression	No	No
*inh*A	Reduced affinity for drug	Reduced fatty acid biosynthesis	Secondary mutation in *inh*A promoter (hypothesis)
*rpo*B	Decreased DNA polymerase affinity for drug	Reduced DNA transcription efficiency	Secondary mutation in *rpo*A, *rpo*C or *rpo*B
*rps*L & *rrs*	Reduced ribosomal target affinity for drug	Impaired ribosome performance, Reduced protein synthesis accuracy	Unknown
*emb*B	Decreased arabinosyl transferase affinity for drug	Reduced cell wall biosynthesis efficiency	Secondary mutation in *emb*ABC operon (hypothesis)
*pnc*A	Reduction or loss of pyrazinamide prodrug activation	Unknown	Unknown
*gyr*A & *gyr*B	Reduced DNA gyrase affinity for drug	Reduced DNA supercoiling, DNA replication and transcription efficiency	Secondary mutation in *gyr*A or *gyr*B (hypothesis)
*Eis*	Aminoglycoside acetyltransferase overexpression	No	No

Almost all laboratory‐generated mutants with a rifampicin resistance‐associated mutation in the RRDR of *rpo*B show a significant fitness deficit compared with their drug‐susceptible ancestors when grown in the absence of this drug. Therefore, it was hypothesized that the fitness cost linked to rifampicin resistance could be reduced by compensatory mutations in clinical isolates (Billington et al., 1999; Comas et al., 2012; Mariam et al., 2004; de Vos et al., 2013). Nonsynonymous mutations in the *rpo*A and *rpo*C genes that encode the α and β’ subunits of RNA polymerase, respectively, could play the role of fitness‐compensatory mutations in rifampicin‐resistant *rpo*B mutants (Comas et al., 2012; de Vos et al., 2013). Indeed, it was reported that part of rifampicin‐resistant isolates with a *rpo*B mutation also carry a nonsynonymous mutation in the *rpo*A or *rpo*C gene in South Africa (27.1%, 89/329), China (27.8%, 89/320) and Korea (39.4%, 67/170) (Comas et al., 2012; Li et al., 2016; Song et al., 2014). In addition, clinical isolates that carry mutations in the RRDR of *rpo*B and also in *rpo*A/*rpo*C display higher competitive fitness in vitro and in vivo compared with laboratory‐generated rifampicin‐resistant mutants that carry only the same *rpo*B RRDR mutation and that belong to the same phylogenetic lineage (Brandis & Hughes, 2013; Comas et al., 2012; Song et al., 2014). These data suggest that mutations in the *rpo*A/*rpo*C genes are fitness‐compensatory mutations that alleviate the costs of *rpo*B mutations. Furthermore, genetic reconstructions in a *Salmonella* model demonstrated that mutations not only in *rpo*A and *rpo*C, but also in *rpo*B are associated with higher growth rate (Brandis & Hughes, 2013; Brandis et al., 2012). In fact, many previous studies showed that rifampicin‐resistant *M. tuberculosis* clinical isolates carry multiple (double, triple and quadruple) mutations in the *rpo*B gene (Bahrmand, Titov, Tasbiti, Yari, & Graviss, 2009; Casali et al., 2014; Nguyen, Nguyen, et al., 2017; Song et al., 2014). This could be the result of compensatory mechanisms to alleviate the fitness cost exerted by specific mutations (Brandis & Hughes, 2013; Brandis et al., 2012). It is worth noting that compensatory mutations are more commonly identified in the dominant MDR, pre‐XDR and XDR clones in high MDR TB burden countries, suggesting that high drug‐resistant mutants harbouring these mutations can be successfully transmitted in human populations (Casali et al., 2014; Cohen et al., 2015; Comas et al., 2012; Klopper et al., 2013; Li et al., 2016; de Vos et al., 2013).

These studies also showed that the *rpo*C mutation is significantly associated with the *rpo*B S531L mutation, suggesting an interaction between a fitness‐compensatory mutation and a specific drug resistance‐associated mutation (Casali et al., 2014; Li et al., 2016; de Vos et al., 2013). This may explain why *rpo*B S531L is the most common mutation observed in rifampicin‐resistant clinical isolates and displays a low biological cost. The presence of compensatory mutations seems to be associated with Beijing strains, especially those harbouring the *rpo*B S531L variant (Casali et al., 2014; Li et al., 2016). Nevertheless, the frequency of compensatory mutations differs according to the Beijing genotype subclades, suggesting epistatic interactions (Box 1, see below) between drug resistance mutations, compensatory mutations and genetic background (Casali et al., 2014).

## 7 ACCUMULATION OF DRUG RESISTANCE‐ASSOCIATED MUTATIONS AND EPISTASIS

### 7.1 Accumulation of drug resistance‐associated mutations

The high diversity of mutations in *M. tuberculosis* suggests different evolutionary trajectories towards highly resistant genotypes, in response to various selection pressures. Nevertheless, for almost all drug resistance‐associated genes, the predominance of some specific mutations, generally known to be associated with high level of resistance and low biological cost, has been described (see Table 2). As a result, combinations of at least two specific mutations, such as *rpo*B531, *kat*G315, *rps*L43, *emb*B306 and *gyr*A94, are favoured (Casali et al., 2014; Cohen et al., 2015; Nguyen, Nguyen, et al., 2017). Although the quality of the treatment undoubtedly plays a role in the emergence of particular drug resistance mutations, the strains with drug resistance‐associated mutations seem to have higher propensity to accumulate other drug resistance mutations in the same gene or in different genes (Bahrmand et al., 2009; Jagielski et al., 2014; Nguyen, Nguyen, et al., 2017; Shen et al., 2007). For instance, the *kat*G315, *emb*B306 or *pnc*A mutations are more frequently observed in MDR than in non‐MDR isolates (Hazbon et al., 2006; Nguyen, Contamin, et al., 2017; Salvatore et al., 2016; Shen et al., 2007). All these data suggest a cumulative effect of mutations that are specifically associated with drug resistance and the occurrence of epistasis (Figure 1c). Besides epistatic interactions, Chang et al. (2015) in their review of the causes of the excess of MDR infections suggest that the associated linkage selection can also be at the origin of the proliferation of multiple drug‐resistant bacteria. This is especially the case for *M. tuberculosis* which follows a basic clonal evolution model (see above). This model generates a strong linkage disequilibrium that may favour the coexistence of two or more particular drug resistance‐associated alleles.

### 7.2 Epistasis between drug‐resistant mutations

Although little is known about epistasis between drug resistance mutations in *M. tuberculosis,* a finding suggests that it could play an important role in the emergence and evolution of MDR and XDR *M. tuberculosis* strains (Borrell et al., 2013). The interaction between drug resistance mutations may restore or even increase the biological fitness of drug‐resistant mutants compared with drug‐susceptible strains. For example, Spies et al. demonstrated that the double mutants *rps*L K43R/*kat*G S315T, *rps*L K43R/*rpo*B S531L and *rpo*B S531L/*kat*G S315T, which are frequently detected in clinical isolates, grow faster than drug‐susceptible strains (Spies et al., 2013). This suggests that the interaction between these mutations may offer a fitness advantage to the double mutants. Indeed, these double mutations increase the fitness of drug‐resistant *E. coli* and drive the evolution of MDR acquisition (Trindade et al., 2009). On the contrary, Salvatore et al. found that isolates that carry the *kat*G S315T/*rps*L K43R mutations are less frequent among MDR strains that cause multiple TB cases in a household than among single‐case household MDR strains, suggesting the occurrence of negative epistasis (Salvatore et al., 2016). As the *kat*G315 mutation does not affect the virulence and transmission of isoniazid‐resistant strains, the authors suggested that the *rps*L43 mutation might impart a biological cost on the transmissibility of drug‐resistant bacteria. Nevertheless, the combination of *kat*G315 and *rps*L43 mutations is common in drug‐resistant clinical *M. tuberculosis* isolates, particularly in Beijing strains. This suggests that epistasis varies according to the strain genetic background (see below). Borrell et al. (2013) described epistatic interactions between mutations associated with resistance to ofloxacin and rifampicin, using *M. smegmatis* as model organism. The authors demonstrated that 35% (6/17) of double mutants carrying specific *rpo*B and *gyr*A mutations associated with rifampicin and fluoroquinolone resistance have a significant higher fitness than the corresponding single drug‐resistant mutants. In particular, the *gyr*A N94G mutation was associated with improved fitness in all double mutants, irrespectively of the *rpo*B mutation. In an interesting manner, the mutation combinations obtained in vitro in *M. smegmatis* correspond to the most common mutations detected among MDR and XDR clinical isolates in high MDR TB burden countries (Borrell et al., 2013; Casali et al., 2014; Comas et al., 2012). These authors also found some double mutants bearing higher biological cost, which can be a sign of negative epistasis. Furthermore, the acquisition of a secondary mutation (linked or not linked to drug resistance) in the same gene, for example *rpo*B, was associated with a reduction of biological cost (Brandis & Hughes, 2013; Song et al., 2014).

### 7.3 Epistasis between drug resistance‐associated mutations and compensatory mutations

The progressively increasing identification of drug resistant, including MDR and XDR isolates without reduction in fitness, suggests the presence of epistatic interactions between drug resistance mutations and compensatory mutations (Figure 1c). Using the *M. smegmatis* model, Song et al. (2014) demonstrated higher growth rates and higher relative fitness in recombinant strains carrying both *rpo*B S531L and *rpo*C F452L or *rpo*C V483G mutations than in strains harbouring only the *rpo*B S531L mutation. However, these interactions have not been investigated in *M. tuberculosis*. Nevertheless, the findings that many (27%–70%) clinical rifampicin‐resistant mutants carry putative compensatory mutations in either *rpo*A or *rpo*C genes support the hypothesis that these two mutation types interact also in *M. tuberculosis* (Casali et al., 2014; Comas et al., 2012; Li et al., 2016; Song et al., 2014). As example, rifampicin‐resistant *M. tuberculosis* strains carrying the *rpo*B S531L mutation are often associated with putative compensatory mutations in the *rpo*A or *rpo*C genes (Casali et al., 2014; Song et al., 2014; de Vos et al., 2013).

Concerning the evolution of MDR strains, clinical and molecular studies suggest that isoniazid resistance, due to the *kat*G S315T mutation, has preceded the emergence of *rpo*B gene mutations leading to the acquisition of rifampicin resistance (Cohen et al., 2015; Gegia, Winters, Benedetti, van Soolingen, & Menzies, 2017; Manson et al., 2017; Salvatore et al., 2016). The combination of *kat*G315 and *rpo*B531 mutations with a rifampicin‐resistant fitness‐compensatory mutation (e.g., *rpo*C mutation) is favoured in clinical MDR isolates, suggesting that these genotypes lead to primary MDR infections. In an important way, the emergence of XDR TB seems to be caused by the transmission of XDR strains directly from person to person rather than by inadequate MDR treatment (Shah et al., 2017). Thus, compensatory evolution and epistasis could play an important role in the emergence and spread of highly resistant strains in the community.

### 7.4 Epistasis between resistance determinants and genetic background

For many bacteria, epistatic interactions have been also described between resistance determinants and their genetic background (Chang et al., 2015). In *M. tuberculosis*, epidemiological and molecular data have shown the emergence and the successful spread of MDR/XDR clones belonging to Beijing or LAM families carrying specific mutations associated with high level of drug resistance and compensatory mutations (Casali et al., 2014; Cohen et al., 2015; Eldholm et al., 2015). Indeed, in Beijing family, it was demonstrated that the biological costs of resistance mutations are smaller than those in other families, or the acquisition of compensatory mutations appears easier, possibly explaining the association between Beijing genotype and MDR (Casali et al., 2014; Gagneux, Long, et al., 2006).

Altogether, the interactions between different drug resistance mutations, between drug resistance mutations and compensatory mutations and between drug resistance mutations and the genetic background underline the key role of epistasis in the evolution of multiple drug resistance in *M. tuberculosis*.

## 8 CONCLUDING REMARKS

As detailed in this review, drug resistance evolution in *M. tuberculosis* is driven by various factors with different effects. The mutation frequency and type can be affected by the drug‐resistant patterns and genotypes. Different mutations can cause different levels of drug resistance and of biological fitness cost even when they are located in the same gene or on the same codon.

The most frequent drug resistance mutations in clinical isolates worldwide are often associated with high levels of resistance and low/no fitness costs, or combined with compensatory mutations to restore the bacterial fitness. This strongly suggests that epistatic interactions influence the evolution of drug resistance in *M. tuberculosis*. Furthermore, all these evolutionary processes, including the basic clonal reproduction mode of *M. tuberculosis*, not only maintain drug‐resistant strains, but also favour their transmission in host populations. Therefore, besides the well‐known extrinsic factors (inadequate treatment regimens, differences in drug pharmacokinetics‐pharmacodynamics, patient adherence, etc.), intrinsic factors, such as compensatory mechanisms, reduced fitness cost, clonal interference, mutation rate and epistasis, also promote the emergence of MDR and XDR strains worldwide.

In an important way, the Beijing lineage is rapidly spreading worldwide. This lineage is associated with MDR TB as well as with high level of drug resistance and fitness‐compensatory mutations (Casali et al., 2014; Manson et al., 2017). This suggests a worrying scenario in which drug resistance evolves towards very fit and highly drug‐resistant genotypes and the successful transmission of deadly drug‐resistant mutants. This could seriously challenge the success of TB control programmes worldwide.

It is unfortunate that, some drug resistance mechanisms remain unclear and many mechanisms of fitness‐compensatory evolution and epistasis have not been investigated in *M. tuberculosis*. More work is needed to increase our knowledge on all the forces that drive drug resistance in *M. tuberculosis* for better controlling the emergence and rapid spread of highly drug‐resistant strains. As suggested by the levels of drug resistance reached globally, we are losing the arms race against bacteria including *M. tuberculosis* (Bañuls et al., 2018). *M. tuberculosis*, as many pathogens, has a complex ecology and evolution and is also evolving and fluctuating through time and space according to local contexts (Bañuls et al., 2015; Comas et al., 2013; Eldholm et al., 2016; Müller, Borrell, et al., 2013; O'Neill et al., 2015; Trauner et al., 2014). For instance, our review underlines that strains carrying multiple drug‐resistant mutations reveal a high ability to acquire other resistances or compensatory mutations by epistatic interactions in reducing the biological cost imposed on the fitness of bacteria. These evolutionary processes suggest that, to limit the drug resistance escalation, molecules acting simultaneously on multiple bacterial targets are urgently needed to replace single‐target drugs that now require to be used in more and more complex combinations (the standard treatment of TB disease is composed by a minimum of four drugs). In addition, the detailed knowledge of evolutionary mechanisms will help develop accurate models to predict the evolution of drug resistance and thus to better control it as underlined by other authors (Lehtinen et al., 2017; Schenk & de Visser, 2013).

## CONFLICT OF INTEREST

None declared.
